# From *h* to *p* efficiently: optimal implementation strategies for explicit time-dependent problems using the spectral/*hp* element method

**DOI:** 10.1002/fld.3909

**Published:** 2014-04-11

**Authors:** A Bolis, C D Cantwell, R M Kirby, S J Sherwin

**Affiliations:** 1 Department of Aeronautics, Imperial College LondonSouth Kensington Campus, London, UK; 2 School of Computing, University of UtahSalt Lake City, UT, USA

**Keywords:** spectral/*hp* element method, hyperbolic problems, discontinuous Galerkin, explicit time-integration methods

## Abstract

We investigate the relative performance of a second-order Adams–Bashforth scheme and second-order and fourth-order Runge–Kutta schemes when time stepping a 2D linear advection problem discretised using a spectral/*hp* element technique for a range of different mesh sizes and polynomial orders. Numerical experiments explore the effects of short (two wavelengths) and long (32 wavelengths) time integration for sets of uniform and non-uniform meshes. The choice of time-integration scheme and discretisation together fixes a CFL limit that imposes a restriction on the maximum time step, which can be taken to ensure numerical stability. The number of steps, together with the order of the scheme, affects not only the runtime but also the accuracy of the solution. Through numerical experiments, we systematically highlight the relative effects of spatial resolution and choice of time integration on performance and provide general guidelines on how best to achieve the minimal execution time in order to obtain a prescribed solution accuracy. The significant role played by higher polynomial orders in reducing CPU time while preserving accuracy becomes more evident, especially for uniform meshes, compared with what has been typically considered when studying this type of problem.© 2014. The Authors. International Journal for Numerical Methods in Fluids published by John Wiley & Sons, Ltd.

## 1. Introduction

High-order spectral/*hp* element methods, utilising element-wise polynomial spaces of order *P ≥*1, are gaining prominence for the efficient discretisation of time-dependent problems. Originally proposed by Patera [1] for the incompressible Navier–Stokes equations, they are being applied to a range of problems such as cardiovascular, separated and geophysical flows [2]. The exponential convergence of the solution with increasing polynomial order results in lower numerical errors for the same number of degrees of freedom when compared with linear finite element methods [3–5]. As a consequence, long time integration can potentially be achieved more accurately and more efficiently than may be possible with traditional low-order methods.

In combination with a discontinuous Galerkin (DG) projection, the spectral/*hp* element method has been widely used for the solution of hyperbolic equations. Initially proposed by Reed and Hill [6] for solving neutron transport problems, it gained popularity because of its ability to preserve phase and amplitude information during time integration, as demonstrated by Sherwin [7], Ainsworth *et al*. [8–10] and De Basabe *et al*. [11,12]. The numerical properties of DG spectral/*hp* element methods for hyperbolic equation solutions have been investigated by Peterson [13], Cockburn and Shu [14,15], Hu and Atkins [16], Warbuton *et al*. [17] and Hesthaven *et al*. [18].

While the numerical properties of DG spectral/*hp* element methods for sufficiently smooth solutions are now widely recognised in the asymptotic limit [19], the choice of discretisation parameters to achieve a given numerical error in the most computationally efficient manner is not as readily understood. Unlike discretisations for linear finite element methods, those for high-order techniques can be considered a function of both mesh element size (*h*) and polynomial order (*P*), which greatly enrich the space of possible spatial discretisations. Furthermore, the element-wise data locality of these methods has the consequence that traditional operator implementation techniques for low-order finite element methods, where elemental matrices are coalesced into a single large sparse global matrix, may not be the most efficient approach when dealing with higher polynomial orders. For example, local operator implementation using an element-by-element approach has been shown to be more computationally efficient in two dimensions [20] on CPUs, with the performance difference being more pronounced in three dimensions [21], when using a continuous Galerkin (CG) projection. Graphics processing units are more efficient when there is limited indirection; hence, the local element-by-element approach is the best choice even for linear finite element methods [22]. Sum factorisation [23] exploits the tensor-product nature of the high-order elemental construction to cast the elemental operations as a sequence of smaller matrix–matrix products, which improves the efficiency still further for very high polynomial orders. As a consequence, understanding the computational efficiency of these different implementation strategies and hardware choices across the space of possible discretisations is non-trivial. In this study, we use the DG projection and thus restrict ourselves to considering the local matrix and sum-factorisation approaches. With knowledge of the most efficient technique with which to apply an operator for a specific polynomial order, one might then ask what the optimal choice of discretisation should be to achieve a given solution accuracy at the minimal computational cost [24]. In this case, runtime is now a function of both mesh element size and polynomial order, and there exists a subspace of possible discretisations that satisfy the error constraint, from which we seek the minimum runtime.

The aim of this paper is to extend these previous studies by identifying general trends for the optimal selection of spatial and temporal discretisations for time-dependent problems. When integrating explicitly in time, the efficiency of the algorithm depends not just on the implementation of the spatial operator and its cost per application but also on the number of time steps needed to reach the desired final time and the cost of each step. The number of time steps is related to the discretisation through the CFL condition, which restricts the size of the time step based on the eigenspectrum of the discretised spatial operator. The stability region of the chosen time-integration scheme must enclose all eigenvalues of this operator to ensure numerical stability.

Given a time-dependent problem to solve with a prescribed accuracy on the final solution, we would like to establish the combination of discretisation parameters, operator implementation and time-integration scheme, which minimises the solution time. It is commonly understood that achieving accurate solutions when integrating over long time periods requires the use of high-order time-integration schemes. However, for shorter time-integration periods, spatial errors may dominate, so it is important to understand when high-order schemes are appropriate and when lower-order schemes will suffice and offer the best performance. In this study, we restrict ourselves to a rotating Gaussian transported under a 2D hyperbolic unsteady linear advection problem on a square domain with upwinded Dirichlet boundary conditions. While this test problem is not necessarily representative of the complexity of typical fluid-flow applications, it is sufficiently non-trivial to establish basic trends that can be applied to other more complex PDE problems and will highlight the most important aspects of the spatial and temporal discretisations.

In Section 2, we give an overview of the spectral/*hp* element method and temporal discretisations used along with the test problem and a description of the CFL control. Results are reported in Section 3, while discussion and general trends are given in Section 4.

## 2. Methods and Formulation

The test problem considered is that of the 2D unsteady advection equation on a [ − 1,1]^2^ domain, in which an off-centred Gaussian function is advected about the origin under a constant rotational divergence-free velocity field **V**. The problem is mathematically expressed as


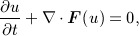
(1a)


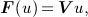
(1b)


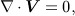
(1c)


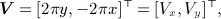
(1d)

with the exact solution for all times *t* given by


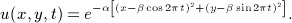
(1e)

The parameters *α* and *β* govern the shape and position of the Gaussian function, respectively. They are fixed at


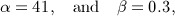


in order to produce a Gaussian function with a standard deviation of *σ* = 0.11, passing through the domain in a prescribed circle of radius 0.3 centred at the origin. The Gaussian function attains a maximum value of O(10^ − 9^) on the domain boundary when the centre passes at its closest point, allowing the use of weakly imposed zero-Dirichlet boundary conditions on all four edges. We explored the impact of the initial condition/boundary condition incompatibility issue; after examination, we concluded that it does not affect the results presented in this paper. The domain is discretised in space using high-order spectral/*hp* elements, which are briefly described in the following section.

### 2.1. Spectral/*hp* element method. 

The spectral/*hp* element method extends the traditional low-order finite element method by adding higher-order polynomial shape functions to each element. As with other finite element methods, a domain Ω is decomposed into a set of non-overlapping elemental regions, Ω_*e*_, such that Ω = ∪ Ω_*e*_. We consider only the case of conformal meshes. Basic operations, such as differentiation or integration, are carried out on a reference element Ω_*st*_, which is mapped to each physical element using an isoparametric coordinate mapping *χ*^*e*^: Ω_*st*_ → Ω_*e*_. In two dimensions, this maps the reference space coordinates (*ξ*_1_, *ξ*_2_) of Ω_*st*_ onto the reference space coordinates (*x*_1_, *x*_2_) as


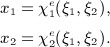


Within the reference space, a variable *u* is approximated via an expansion in terms of a set of *N* two-dimensional basis functions {*φ*_*n*_(*ξ*_1_,*ξ*_2_)}. These functions can be constructed as a tensor product of two sets of *P* + 1 one-dimensional basis functions {*ψ*_*p*_(*ξ*_1_)}and {*ψ*_*q*_(*ξ*_2_)}, where *n* = *n*(*p*,*q*), such that


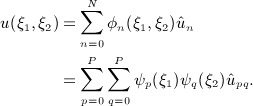


The bases {*ψ*_*p*_(*ξ*_1_)}and {*ψ*_*q*_(*ξ*_2_)}each span the polynomial space of order *P*, and in what follows, we employ hierarchical modal functions. Typically, we choose to use the integral of Legendre polynomials (or the 1,1 Jacobi polynomials), P_*p*_^1,1^(*ξ*), modified in such a way that


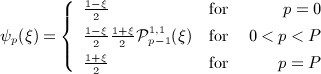


to allow a boundary-interior partitioning of elemental modes.

We now apply the method of weighted residuals with a Galerkin projection. We derive a weak formulation of our problem by multiplying Equation (1a) by smooth test functions, *v*, and integrating over Ω to arrive at


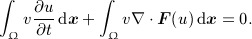


Defining 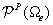 as the space of polynomials of order *P*, the discrete approximation 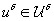 of the variable *u* and the discrete approximations of test functions 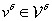 where


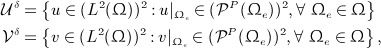


we arrive at the equivalent discrete weak formulation,


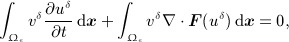
(3)

from which a matrix system can be constructed [3].

For the DG method, we require a mechanism for information to propagate across element boundaries without affecting the stability of the method. Applying the divergence theorem to the second integral of Equation 3, we obtain


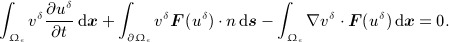
(4)

The coupling is therefore achieved through the boundary fluxes represented by the second integral in Equation 4. The approach used to calculate these fluxes dictates the stability of the method. In this study, we use an up-wind scheme. Defining *u*_ − _^*δ*^ to be the value of the solution *u*^*δ*^ on the boundary of a given element *e* and *u*_ + _^*δ*^ to be the solution on the same boundary of an adjacent element, the boundary flux, denoted with ***f* ƒ **^*e*^(*u*_ − _^*δ*^,*u*_ + _^*δ*^), is defined as


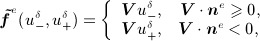


where ***n***^*e*^ denotes the outward-pointing normal to the element. For more details concerning continuous and DG formulations and for the case of more complicated hyperbolic problems (where it may be necessary to use an approximated Riemann solver), see [3,25].

### 2.2. Domain discretisation

**Figure 1:**
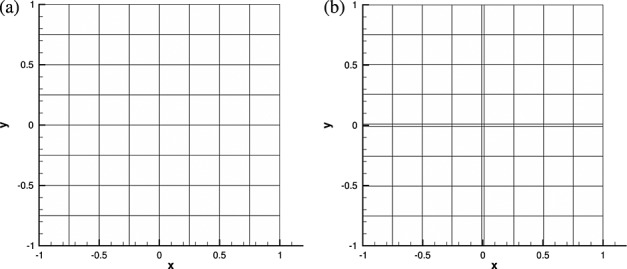
Examples of test meshes used in the study. A uniform mesh with 64 elements (a) and the equivalent non-uniform mesh (b) with 81 elements. The non-uniform mesh includes a narrow cross of elements in the centre of the mesh.

The domain Ω = [ − 1,1]^2^ is discretised using a range of quadrilateral meshes of both a uniform nature and a non-uniform nature. Uniform meshes are structured regular grids of *N* × *N* elements, where *N* is in the range 1, … ,8. An example is shown in Figure 1(a) for *N* = 8. We also consider five non-uniform meshes that contain a mixture of small and large elements. The inclusion of small elements in some part of the domain aims to reproduce the numerical effects arising in practical problems where mesh refinement is used to capture features of the solution (e.g. boundary layer refinement). For these, we take the four uniform meshes where *N* is even and add a narrow vertical and horizontal band of elements of width *h* = 0.01 in the centre of the mesh, an example of which is shown in Figure 1(b). Although this mesh contains 81 elements, for the purpose of comparison, we denote this mesh as being *non-uniform N* = 8 because it is approximately equivalent to the *N* = 8 uniform mesh.

**Figure 2:**
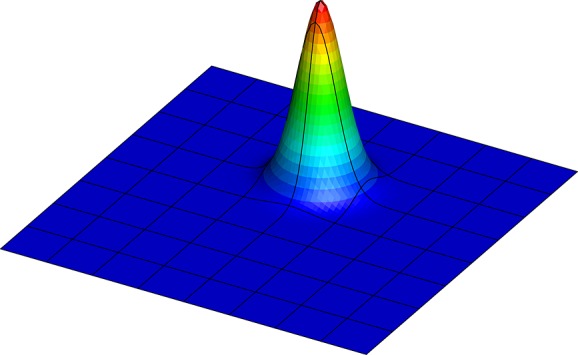
A 2D unsteady advection problem, initial condition projected on 64 uniform elements with *P* = 11.

The Gaussian function given in Equation (1e) at *t* = 0 is projected onto each mesh and used as an initial condition for the simulation. An example is shown in Figure 2 and is the discretised form of the exact solution on the mesh shown in Figure 1(a) with *P* = 11.

### 2.3. Time-discretisation and CFL control

In this study, the temporal derivative is discretised using three explicit time-integration schemes. These can generally be described using the Butcher tables [26] and may be implemented in a unified fashion, as detailed in [27]. The first method is the multi-step second-order Adams–Bashforth (AB2) scheme. We also consider both the second-order and fourth-order explicit Runge–Kutta schemes (RK2 and RK4) described by the following Butcher tables:


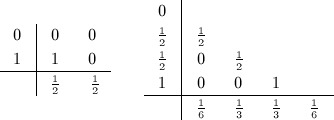
(5)

In general, the stability of an explicit time-integration scheme is governed by the CFL condition [28]. For spectral methods, this has been investigated by various authors (see, e.g. Gottlieb and Tadmor [29]). Analytical descriptions of the stability properties of the DG spectral/*hp* element method when associated with an explicit time-stepping scheme are also available as reported by Zhang and Shu [30] and, more recently, by Antonietti *et al*. [19].

We express the semi-discrete system in Equation 3 in terms of the coefficients as


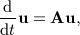
(6)

where **A** represents the discretisation of the linear advection operator and **u** is the vector of expansion coefficients. To maintain numerical stability, the eigenvalue spectrum of **A** must lie within the stability region of the chosen time-integration scheme. Therefore, the CFL condition becomes more stringent as the magnitude of the eigenvalues of **A** increases.

Attempts have been made to understand the behaviour of the eigenspectrum for spectral/*hp* element methods with respect to changes in the discretisation [16]. Sherwin *et al*. [7] investigated (semi-analytically) the behaviour of the 1D hyperbolic equation, discretised with CG and DG methods, showing that discontinuous projections have significant damping effects at high frequencies. Karniadakis and Sherwin [3] indicated a growth rate of the maximum eigenvalue proportional to *P*^2^ for 2D meshes, both for CG and DG projections. Warburton performed a study to understand the trend of the eigenvalues for 2D hyperbolic problems and DG projections; his studies showed similar results [31] and presented optimal numerical techniques to alleviate the eigenvalues’ growth [17].

**Figure 3:**
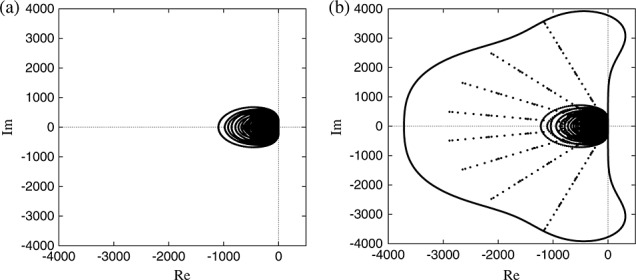
Eigenvalue distributions with *P* = 7 for the (a) isotropic mesh shown in Figure 1(a) and (b) the anisotropic mesh shown in Figure 1(b). For the anisotropic case, the stability region for the fourth-order Runge–Kutta scheme is shown, scaled to encompass the eigenvalue distribution.

For each of the test cases considered in this study, the full eigenspectrum of the weak advection operator **A** has been calculated using LAPACK [32]. Figure 3 shows examples of the eigenvalue spectra for uniform and non-uniform meshes at *P* = 7. While the eigenvalue distribution may show a predictable trend, as discussed earlier, we use the values calculated by LAPACK for implementing the CFL condition, computing for each numerical simulation the restriction on Δ*t* as


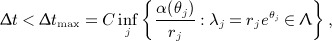
(7)

where *C* is the desired CFL number (generally 0 < *C ≤*1), Λ is the eigenspectrum of the discrete spatial operator **A** and *α*(*θ*_*j*_) denotes the distance from the origin of the boundary of the stability region along the azimuthal of the *j*th eigenvalue. The value of Δ*t* can be interpreted as rescaling the stability region of the time-integration scheme. The bound imposed by Δ*t*_max_ ensures that the stability region is necessarily large enough to enclose all the eigenvalues of **A**. In Figure 3(b), we also show the stability region of the fourth-order Runge–Kutta scheme, scaled by Δ*t*_max_ to minimally enclose the eigenvalue distribution of the spatial operator constructed on the non-uniform mesh in Figure 1(b). As is apparent in the figure, the eigenvalues that are in closest proximity to the boundary of the rescaled stability region of the scheme may not necessarily be those having maximum modulus or real part, because of the shape of the stability region itself.

Analysing deformed variants of the presented isotropic discretisations, where curvatures appear, our investigations showed that the overall shape of the eigenspectrum does not change significantly. The main effect of deformation is an increase of the eigenvalues’ magnitude. In fact, in proximity of deformed edges, the local *h* value is proportionally reduced as the deformation becomes more accentuated. In these cases, a further reduction of Δ*t*_max_ is required to preserve numerical stability. From a computational point of view, deformations act to increase the overall computational time. This is because additional operations are required to handle curved edges and because more time steps are needed to reach the desired final time. While the overall computational time is greater, the general trends do not differ from the non-deformed test cases we presented.

## 3. Results

In order to better understand which aspects of the spatial and temporal discretisations lead to errors in the solution, along with their relative contribution, we introduce the following model to describe the total error:


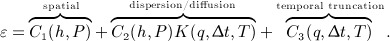
(8)

Equation 8 is composed of three terms, denoting different sources of error, and the simulations outlined in the remainder of this section aim to assess the relative contributions of each of these throughout the parameter space. The first term, *ε*_*p*_ = *C*_1_(*h*,*P*), represents the projection error, that is the contribution due to the projection of the initial condition onto the discrete space. This term is time independent and occurs once at the beginning of the time integration; it is therefore only a function of the discretisation. The third term *ε*_*t*_ = *C*_3_(*q*,Δ*t*,*T*) is the truncation error introduced when discretising the temporal derivative. This error is not directly dependent on the chosen spatial discretisation but depends on the order of the time-integration scheme used (indicated by *q*), the time step Δ*t* and the final time, *T*. The remaining term accounts for the dispersion/diffusion error of the method and numerical errors associated with multiple applications of the spatial operator. This term couples the spatial and temporal discretisations, where *K*(*q*,Δ*t*,*T*) is the number of applications of the spatial operator, which may vary from scheme to scheme, as well as due to the size and number of time steps taken.

### 3.1. Test system

We present results obtained through numerical experiments. The simulations have been run in serial on a 64-bit Mac Pro (Apple Inc, San Jose, California) using a 2.26-GHz Quad-Core Intel Xeon E5520 processor (8 MB of L3 cache) and 16 GB of RAM. The operating system was OSX with a 10.8 Darwin kernel. All tests were performed using the *Nektar++* spectral/*hp* element framework version 3.1.0 [33], which provides the various operator implementations and time-integration schemes within a common software package to ensure straightforward comparison of the results. The Accelerate Framework provided with OSX was used for BLAS operations.

### 3.2. Projection error *ε*_*p*_. 

**Figure 4:**
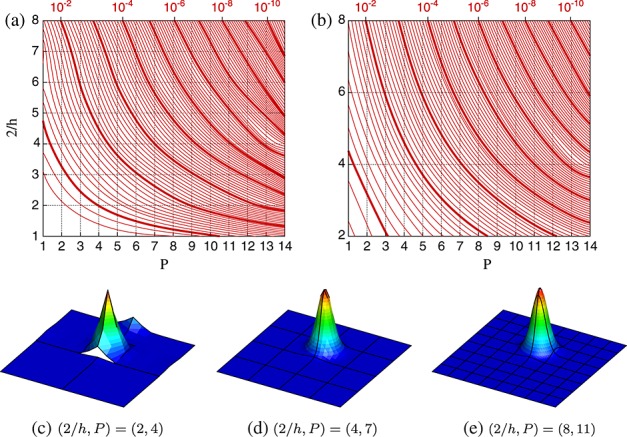
*L*^2^ projection error, *ε*_*p*_, of the initial Gaussian function onto spectral/*hp* discretisations of a [ − 1,1]^2^-domain using (a) uniform meshes and (b) non-uniform meshes. Gridline intersections indicate possible (*h*,*P*) discretisations. Examples of the solution with uniform meshes for different levels of accuracy are also provided for (c) *ε*_*p*_ = 10^ − 1^, (d) *ε*_*p*_ = 10^ − 3^ and (e) *ε*_*p*_ = 10^ − 8^.

The first source of error in all tests is the projection error introduced when the infinite-dimensional initial function is projected onto the finite-dimensional discrete space through a DG approximation. This error is computed as *ε*_*p*_ = | | **u** − **u**^*δ*^ | |  *L*_2_, where **u** and **u**^*δ*^ denote the analytic function and discrete representation, respectively. This is depicted in Figure 4, which shows the error for both uniform and non-uniform meshes. The format of these plots shows increasing number of elements 2  *h* on the *y*-axis, with increasing polynomial order *P* of the expansion used on each element along the *x*-axis. Although the data are discrete, we plot them in a continuous form for the benefit of analysis. Here, *h* corresponds to the size of each element in both coordinate directions. The isolines denote constant *ε*_*p*_, where bold lines denote orders of magnitude. This notation will be used throughout the remaining figures in this paper to represent constituents of the solution error. For highly refined discretisations, projection errors may be as low as *ε*_*p*_ = 10^ − 10^. Below an error of 10^ − 3^, it can be seen that doubling the polynomial order decreases the error by a significantly greater magnitude than doubling the number of elements. This highlights the improved convergence properties of high-order discretisations.

For non-uniform meshes, *y*-axis values are set to correspond to the uniform mesh they approximate. For example, a non-uniform mesh with 81 elements corresponds to a uniform mesh of 64 elements with the additional 17 elements arising from the narrow strips of elements in the centre of the mesh and would be represented by 2  *h* = 8 on the non-uniform plots, as can be seen in Figure 1. The coarsest non-uniform mesh consists of nine elements, corresponding to the four-element uniform mesh. There are few differences in the magnitude of the projection error on non-uniform meshes in comparison with the uniform equivalents. The only notable difference is for few elements and low polynomial order where the narrow elements provide a noticeable increase in projection accuracy.

### 3.3. Effect of time-integration schemes

We now investigate how the choice of time-integration scheme affects the *L*_2_ error. Additionally, for each of the three schemes, we will consider two durations of integration in order to help assess when the error introduced by a given scheme becomes important. Short time integration is understood to be integration to a final time of *T* = 0.25, corresponding to the Gaussian being advected for a quarter of a rotation around the domain and equivalent to a distance of approximately two widths of the bump. Long time integration equates to integration to a final time of *T* = 4.00, corresponding to four cycles around the domain and therefore approximately 32 wavelengths.

**Figure 5:**
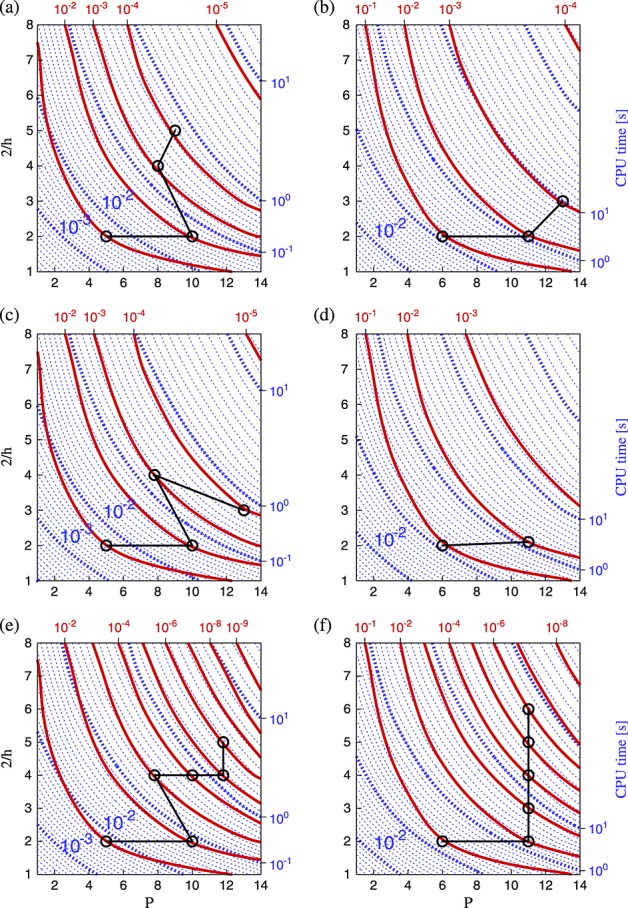
Isolines of *L*_2_ error (solid red) and CPU time (dotted blue) for second-order Adams–Bashforth (a, b), second-order Runge–Kutta (c, d) and fourth-order Runge–Kutta (e, f), at times *T* = 0.25 (a, c, e) and *T* = 4.00 (b, d, f). All plots are for uniform meshes using the local matrix operator implementation. Black circles denote the optimal (*h*,*P*) discretisation for the contours of error where the minimum lies within the explored parameter space.

Figure 5 summarises these tests for uniform meshes using the local elemental matrix approach for operator evaluations. The left column of plots in this figure correspond to short time integration, while the right column shows results for long time integration. The contours of error now correspond to the total error *ε* accumulated throughout the simulation. In addition, we overlay contours of CPU time. We measure only the time-integration portion of the total execution, discounting setup costs and I/O. Given a prescribed error tolerance, one now seeks to find a discretisation that achieves this tolerance in the minimal CPU time. This corresponds precisely to the (*h*,*P*) combination of minimal runtime, which lies on, or to the right of, the chosen error contour. Such minima are denoted by black connected circles, highlighting the optimal path to follow to reduce error at minimal computational cost.

The first observation is that while solution accuracy is comparable across all time-integration schemes on coarse meshes, the fourth-order Runge–Kutta scheme achieves far greater accuracy on finer meshes than the second-order schemes. Integrating over long time periods leads to a greater relative increase in error for refined meshes than for coarse meshes across all time-integration schemes. These two regimes correspond to where temporal and spatial errors dominate; this will be explored more precisely in Section 3.6.

**Figure 6:**
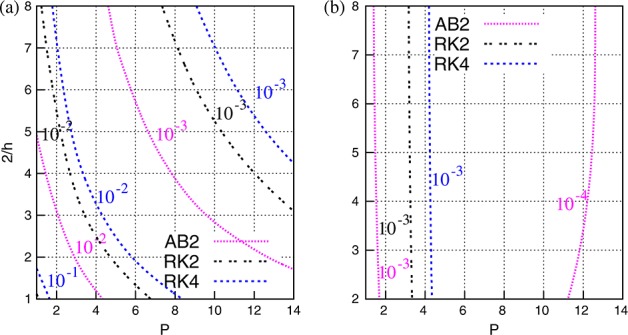
Maximum time step as dictated by the CFL constraint for (a) uniform and (b) non-uniform meshes using second-order Adams–Bashforth, and second-order and fourth-order Runge–Kutta schemes.

CPU time clearly increases with longer time integration. The time step used in each test is chosen at the limit of the CFL condition, *C* = 1, and is reported in Figure 6. The choice of *C* derives from the assumption that we do not have *a priori* knowledge of the initial condition, and therefore, all eigenvectors could potentially be energised. While Figure 6(a) shows that Δ*t*_max_ clearly depends on both *h* and *P* for uniform meshes, Figure 6(b) highlights that for non-uniform meshes, the maximum time step is almost independent of *h* for the parameter space considered. It is apparent that for uniform meshes, Runge–Kutta schemes support a larger time step than Adams–Bashforth. For example, for *P* = 8 and 2  *h* = 4, the second-order Adams–Bashforth scheme requires a time step  10^ − 3^, while the fourth-order Runge–Kutta scheme requires only  10^ − 2.5^. However, the fourth-order Runge–Kutta scheme supports only a slightly larger time step than its second-order counterpart, particularly on coarse meshes.

From the contours in Figure 5, we note that for highly accurate solutions, the only feasible strategy is to use a high-order discretisation and a high-order time-integration scheme together to reduce projection and temporal truncation errors. Even if larger time steps can be used with the fourth-order Runge–Kutta scheme, it remains slightly more computationally expensive overall than the second-order version because each step requires more work per time step. Therefore, if we have a high tolerance of errors (e.g. 10^ − 1^), a second-order time-integration scheme using a lower-order discretisation obtains the result in less time than a higher-order scheme, even for the long time period investigated.

We now highlight those (*h*,*P*) combinations that achieve the lowest runtime for each order of magnitude in solution error. These optimal discretisations do not show a clear pattern, but in general, to achieve a more accurate solution over long times with second-order time-integration schemes, the trend suggests that increasing polynomial order offers the most effective strategy. This makes sense, because dispersion errors from repeated application of the operators will decrease exponentially with increasing *P*. For short times, the total error has a lower temporal component, so a more balanced increase in mesh refinement and polynomial order gives the best performance by reducing projection error (i.e. moving normal to the contours of *ε*_*p*_). The fourth-order scheme suggests that for long time periods, increasing mesh element density (*h* refinement) is the best approach, but such a conclusion may be considered misleading because the CPU time and error contours are essentially parallel in this region of the parameter space.

### 3.4. Non-uniform meshes

**Figure 7:**
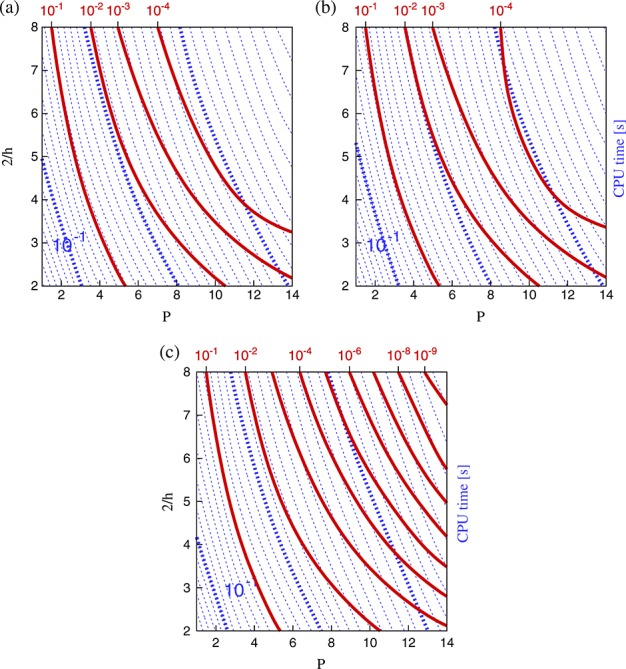
Isolines of *L*_2_ error (solid red) and CPU time (dotted blue) for second-order Adams–Bashforth (a) second-order Runge–Kutta (b) and fourth-order Runge–Kutta (c) at time *T* = 4.00 for non-uniform meshes using the local matrix approach. These correspond to the uniform mesh plots in Figure 5(b, d, f), respectively.

Introducing non-uniformity into the mesh has the most apparent effect on coarse meshes where the small elements impose a much stronger restriction on the CFL limit, and therefore the time step, than would otherwise be the case. This is shown in Figure 7(a–c), where CPU time is significantly higher for coarse discretisations than in the equivalent plots for uniform meshes in Figure 5(d, e, f). The increase is less pronounced on finer meshes because the disparity of element sizes is reduced. As a consequence of this change, the choice of optimal discretisation on non-uniform meshes is typically in the fine-mesh, low-order range. In contrast to the uniform case, to improve accuracy in the solution, the best strategy for non-uniform meshes is to increase mesh refinement. For smaller error tolerances, Figure 7 suggests increasing *P* is the optimal strategy; however, this is purely an artificial consequence of the finite bounds imposed on the parameter space of this study. It should be noted that, even at *ε* = 10^ − 2^, the discretisation giving minimum CPU time uses *P*4, which is significantly higher than most conventional finite element methods.

### 3.5. Operator implementation

**Figure 8:**
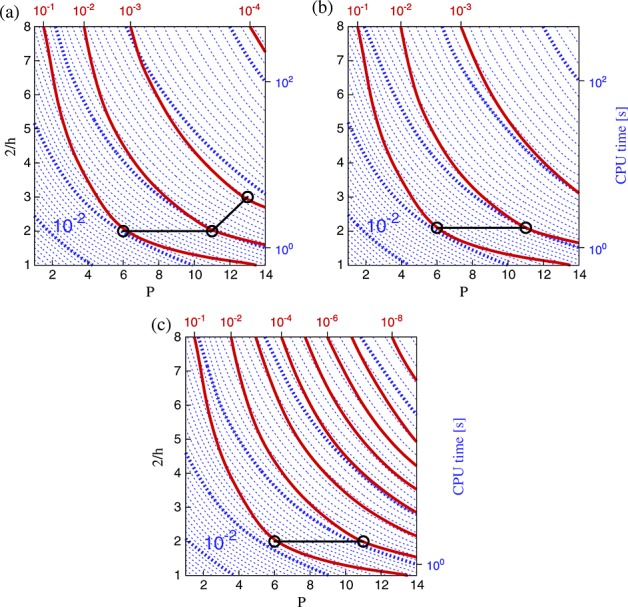
Isolines of *L*_2_ error (solid red) and CPU time (dotted blue) for second-order Adams–Bashforth (a), second-order Runge–Kutta (b) and fourth-order Runge–Kutta (c) at time *T* = 4.00 for uniform meshes using the sum-factorisation approach. These correspond to the local matrix approach plots in Figure 5(b, d, f), respectively. Black circles denote the optimal (*h*,*P*) discretisation for the contours of error, where the minimum lies within the explored parameter space.

So far, we have only assessed performance using the local elemental matrix approach for performing matrix–vector multiplication. In this case, applications of the explicit matrix operators are performed using a block-diagonal matrix, where each block corresponds to the operator on a single element of the domain. This was shown to be efficient in the continuous Galerkin case for intermediate polynomial orders (*P*  4 to *P*  7), while at higher polynomial orders, sum factorisation is found to be more efficient [20,21,24]. In Figure 8, we present timings for uniform meshes and the sum-factorisation technique. These confirm that the findings in the literature are also valid for the DG case. Furthermore, the optimal discretisations for all error tolerances now lie in the coarse-mesh, high-order regime, because this is the parameter range in which the technique is most efficient. Discussion of this aspect is covered in the literature, so we do not consider it further here.

### 3.6. Spatial/temporal dominance

**Figure 9:**
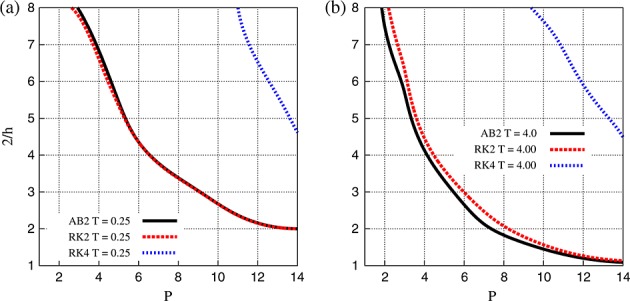
Influence zones for uniform meshes and the three time-integration schemes considered for (a) short time integration and (b) long time integration. Lines indicate *κ*  *ε* = 1, where *κ* corresponds to the error when using *C* = 0.1. Discretisations where the spatial error dominates are to the lower left of the line, while to the upper right, temporal error dominates.

To further understand the relative contributions of the remaining terms in Equation 8, we measure the error, *κ* in the solution when using a CFL constant of *C* = 0.1. This has the effect of reducing the time step by an order of magnitude, and consequently, we can consider the truncation error, *κ*_*t*_ = *C*_3_(*q*,Δ*t*,*T*) to be small or negligible. The remaining error arises from the projection error, *κ*_*p*_ ≡ *ε*_*p*_, and the dispersion error, *κ*_*d*_ < *ε*_*d*_, introduced through the repeated application of the spatial operators. This enables us to identify for which discretisations the ratio of *κ*  *ε*  1, where we recall that *ε* is the error with *C* = 1. For *κ*  *ε* > 1, we have that the spatial error is dominating and where *κ*  *ε* < 1 temporal errors dominate. Figure 9 summarises these data for the three time-integration schemes. The lines indicate the boundary between the spatial and temporal error dominance. The region to the left of a given line indicates discretisations for which the dominant error is due to spatial inaccuracy, while the region to the right corresponds to temporal error dominating. As expected, the error from using fourth-order Runge–Kutta is predominantly spatially dominant unless refined high-order discretisations are used. This is consistent with the earlier analysis, indicating that one should increase *P* for optimal execution time given a desired accuracy. For both second-order schemes, the break-even point occurs with much coarser discretisations. Over longer time integration, the region of temporal dominance extends further towards coarser meshes and lower polynomial orders. This is a consequence of the additional dispersion error introduced by the order of magnitude increase in the number of time steps taken to reach the same final time. Although the spatial/temporal dominance is qualitatively predictable, it is interesting to remark how those regions are actually shaped in the (*h*,*P*) plane and where their boundaries are located for the specific case.

### 3.7. Performance prediction

The ability to predict the time required for a simulation depends on the accuracy when forecasting the eigenvalue distribution of the weak advection operator, given that a direct calculation is often prohibitive in real applications. In order to enhance the understanding of the CFL restrictions that govern our simulations, we investigate the spectrum of **A** for regular meshes. Our intention is to recognise a trend in the growth rate of the eigenvalues with respect to (*h*,*P*) and then predict Δ*t*_max_ using Equation 7.

We assess this by monitoring, during our numerical experiments, the magnitude | *λ*_*dom*_ | of the eigenvalue that dominates the stability of the scheme. For regular meshes, the eigenvalue that quantifies the CFL restriction appears to be the one showing the minimum real part, that is, *θ*_*j*_ = *π*. We model | *λ*_*dom*_ | growth rate as


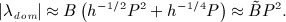
(9)

**Figure 10:**
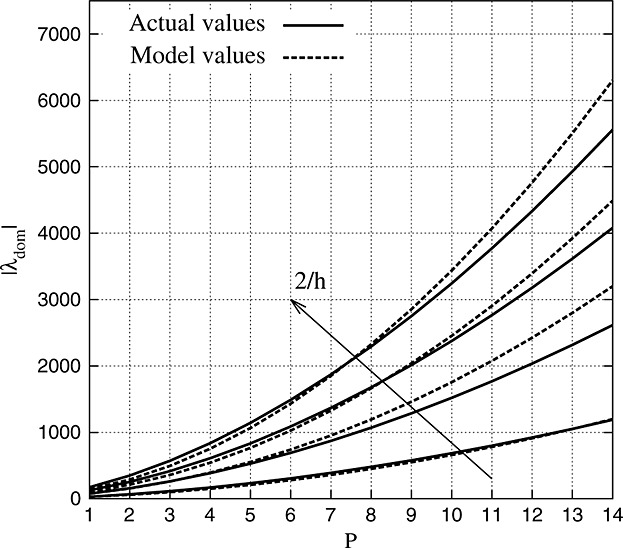
Dominant eigenvalue magnitude for uniform meshes. Actual values obtained using LAPACK (solid lines) are compared with the estimate (dashed lines) of Equation 9.

Throughout a calibration process, we extract *B* = 9.6265. Figure 10 shows a comparison between the actual values of | *λ*_*dom*_ | and the model predictions. Although Equation 9 is a rough estimate of | *λ*_*dom*_ | , the discrepancies between the forecasted and actual values are always less than 20*%*. The maximum error appears for high values of *P*, where the model overestimates the eigenvalue magnitude.

The model reported in Equation 9, although problem specific, is consistent with what is anticipated in [3], where | *λ*_*dom*_ | growth rate was identified as being proportional to *P*^2^ for a weak advection operator. Provided that Δ*t*_max_ can be estimated using Equations 9 and 7, we can know beforehand the CPU time required for a specific (*h*,*P*,*T*) combination.

## 4. Conclusions

We have systematically assessed the relative importance of discretisation and time-integration scheme when targeting minimal runtime. The spatial discretisation and time-integration schemes both impose restrictions on the overall accuracy of the solution, but their relative error contributions will vary depending on the exact choice of discretisation parameters chosen. All the results demonstrate that there are substantial benefits for using high-order methods for transient problems while also highlighting some of the subtleties in choosing optimal discretisations to minimise runtime.

For each time-integration scheme and specific choices for the final time *T*, we have identified the region in the (*h*,*P*) plane for which the error in the solution is primarily due to the underlying inaccuracy of the spatial discretisation rather than a consequence of time integration. Outside this region, typically for more refined discretisations, time-integration errors are the dominant cause of solution error. These divisions naturally differ for the three time-integration schemes with the spatially dominant zone extending to finer discretisations for high-order time-integration schemes, compared with the lower-order counterparts. A consequence of this is that higher-order time-integration schemes offer no advantage over their computationally less-expensive lower-order counterparts if the solution error at the chosen discretisation is spatially dominated under both schemes.

The choice of the time-integration scheme therefore requires careful consideration. In particular, we have noted that for short time integration and for error tolerances down to 10^ − 3^, high-order time-integration schemes, such as the fourth-order Runge–Kutta, are not competitive for our 2D advection test problem. Second-order Adams–Bashforth and second-order Runge–Kutta achieve the same solution accuracy in lower runtimes in these cases. However, achieving highly accurate solutions typically requires a high-order discretisation, and therefore a high-order time-integration scheme, in order to keep both the spatial and temporal error contributions sufficiently small. Furthermore, over long time-integration periods, the shift in the break-even point between spatially and temporally dominated zones dictates that high-order time-integration schemes are more important for maintaining overall solution accuracy.

High-order methods offer exponential reduction in error with increasing polynomial order. Increasing *P* should therefore offer a more attractive approach to increasing the solution accuracy than refining the mesh. This is evident in some of the uniform mesh results, particularly for long time-integration periods. The second-order schemes show significant variation in CPU time along a given error contour, and the path of minima is predominant in the direction of increasing *P*.

In changing the elemental polynomial order, the choice of implementation strategy for matrix–vector operations requires consideration. For continuous Galerkin, the literature highlights the use of a whole-domain global matrix approach for low polynomial orders, a local elemental block-matrix approach for intermediate orders and the local elemental sum-factorisation approach for higher orders. The exact break-even points between these different strategies is, of course, dependent on the element type and performance of the computational hardware, but general observations can be made. We confirm a similar trend is true for DG projections for the local elemental and sum-factorisation strategies.

We conclude with a discussion of the effect of element-size diversity on the time step and consequently the selection of spatial and temporal discretisations for optimal performance. Variation in size and advection velocity across mesh elements dictates the spread of the eigenspectrum of the spatial operator, with smaller size-to-velocity ratios leading to greater magnitude eigenvalues. This leads to a more restrictive time step in order to enclose the entire eigenspectrum inside the stability region of the time-integration scheme. In general, accuracy on uniform meshes can be best achieved using a high-order discretisation and best improved through further increasing the polynomial order. While high-order discretisations are still effective for the non-uniform meshes considered, the most efficient way to increase accuracy is to reduce the size of the larger elements, thereby essentially converging towards a uniform mesh. This aligns with the common wisdom that *h* refinement is most appropriate on meshes where there is a significant disparity in element size. Furthermore, it also raises the possibility of introducing variable polynomial orders and/or non-conforming meshes, which were not explored in this study. While these spatial discretisation features are of extreme interest for practical applications, they would shift the focus of our analysis. In fact, they would introduce a further level of optimisation, as we would seek the optimal (*h*(*x*,*y*),*P*(*x*,*y*)) combination for each specific test case, thus making the analysis too intricate.

Finally, there is a common understanding that high-order methods generally lead to stringent CFL limitations, because of the eigenvalues of the spatial operators grow as a polynomial power of *P*. However, we have shown that even for a coarse error tolerance on the solution, high-order methods often become the most efficient choice. This is due to the accuracy of the solution increasing faster than the stability requirements limit the time step. Consequently, high-order methods offer substantial performance over their linear-order counterparts for transient simulations.

### 4.1. Limitations

As we stated from the outset, the absolute numerical values presented in this paper are code dependent and will also vary along with the nature of the problem, size of the problem and the machine used. However, the numerical experiments highlight some general trends, and the results support the common wisdom that high-order methods are particularly important for long and accurate time integration. In addition, the analysis presented is confined to 2D discretisations; thus, we cannot immediately infer that our considerations can be extended to 3D domains. However, the tensorial nature of the quadrilateral discretisation suggests that similar trends can possibly be observed also for 3D hexahedral discretisations, although further investigations are clearly required to corroborate this.

## References

[1] Patera AT (1984). A spectral element method for fluid dynamics: laminar flow in a channel expansion. Journal of Computational Physics.

[2] Sherwin SJ, Blackburn HM (2005). Three-dimensional instabilities and transition of steady and pulsatile axisymmetric stenotic flows. Journal of Fluid Mechanics.

[3] Karniadakis G, Sherwin SJ (2005). Spectral/hp Element Methods for CFD.

[4] Gottlieb D, Orszag SA (1983). Numerical Analysis of Spectral Methods: Theory and Applications.

[5] Canuto C, Hussaini MY, Quarteroni A, Zang TA (2007). Spectral Methods: Evolution to Complex Geometries and Applications to Fluid Dynamics.

[6] Reed W, Hill T (1973).

[7] Sherwin S (2000). Dispersion analysis of the continuous and discontinuous Galerkin formulation. Lecture Notes in Computational Science and Engineering: Discontinuous Galerkin Methods.

[8] Ainsworth M (2004). Dispersive and dissipative behaviour of high order discontinuous Galerkin finite element methods. Journal of Computational Physics.

[9] Ainsworth M (2004). Discrete dispersion relation for hp-version finite element approximation at high wave number. SIAM Journal on Numerical Analysis.

[10] Ainsworth M, Monk P, Muniz W (2006). Dispersive and dissipative properties of discontinuous Galerkin finite element methods for the second-order wave equation. Journal of Scientific Computing.

[11] De Basabe JD, Sen MK, Wheeler MF (2008). The interior penalty discontinuous Galerkin method for elastic wave propagation: grid dispersion. Geophysical Journal International.

[12] De Basabe JD, Sen MK (2010). Stability of the high-order finite elements for acoustic or elastic wave propagation with high-order time stepping. Geophysical Journal International.

[13] Peterson TE (1991). A note on the convergence of the discontinuous Galerkin method for a scalar hyperbolic equation. SIAM Journal on Numerical Analysis.

[14] Cockburn B, Shu CW (1998). The local discontinuous Galerkin method for time-dependent convection–diffusion systems. SIAM Journal on Numerical Analysis.

[15] Cockburn B, Shu CW (2001). Runge–Kutta discontinuous Galerkin methods for convection-dominated problems. Journal of Scientific Computing.

[16] Hu F, Atkins H (2002). Eigensolution analysis of the discontinuous Galerkin method with nonuniform grids. Part I: one space dimension. Journal of Computational Physics.

[17] Warburton TC, Hagstrom T (2008). Taming the CFL number for discontinuous Galerkin methods on structured meshes. SIAM Journal on Numerical Analysis.

[18] Hesthaven JS, Warburton TC (2008). Nodal Discontinuous Galerkin Methods: Algorithms, Analysis, and Applications.

[19] Antonietti PF, Mazzieri I, Quarteroni A, Rapetti F (2012). Non-conforming high order approximations of the elastodynamics equation. Computer Methods in Applied Mechanics and Engineering.

[20] Vos PEJ, Sherwin SJ, Kirby RM (2010). From h to p efficiently: implementing finite and spectral/hp element methods to achieve optimal performance for low-and high-order discretisations. Journal of Computational Physics.

[21] Cantwell CD, Sherwin SJ, Kirby RM, Kelly PHJ (2011). From h to p efficiently: strategy selection for operator evaluation on hexahedral and tetrahedral elements. Computers & Fluids.

[22] Markall GR, Slemmer A, Ham DA, Kelly PHJ, Cantwell CD, Sherwin SJ (2013). Finite element assembly strategies on multi-core and many-core architectures. International Journal for Numerical Methods in Fluids.

[23] Orszag SA (1980). Spectral methods for problems in complex geometries. Journal of Computational Physics.

[24] Cantwell CD, Sherwin SJ, Kirby RM, Kelly PHJ (2011). From h to p efficiently: selecting the optimal spectral/hp discretisation in three dimensions. Mathematical Modelling of Natural Phenomena.

[25] Zienkiewicz OC, Taylor RL, Sherwin SJ, Peiro J (2003). On discontinuous Galerkin methods. International Journal for Numerical Methods in Engineering.

[26] Butcher JC (2006). General linear methods. Acta Numerica.

[27] Vos PEJ, Eskilsson C, Bolis A, Chun S, Kirby RM, Sherwin SJ (2011). A generic framework for time-stepping partial differential equations (PDEs): general linear methods, object-oriented implementation and application to fluid problems. International Journal of Computational Fluid Dynamics.

[28] Hirsch C (2007). Numerical Computation of Internal and External Flows: Introduction to the Fundamentals of CFD.

[29] Gottlieb D, Tadmor E (1991). The CFL condition for spectral approximations to hyperbolic initial-boundary value problems. Mathematics of Computation.

[30] Zhang Q, Shu CW (2010). Stability analysis and a priori error estimates to the third order explicit Runge–Kutta discontinuous Galerkin Method for scalar conservation laws. SIAM Journal on Numerical Analysis.

[31] Warburton TC (1999).

[32] (2012). http://www.netlib.org/lapack.

[33] (2012). http://www.nektar.info.

